# Conflicto armado, contaminación y riesgos en salud: una evaluación de riesgo de tres fuentes de exposición ambiental asociadas con el conflicto en Colombia

**DOI:** 10.7705/biomedica.5928

**Published:** 2021-12-15

**Authors:** Fabián Méndez, Andrés Mauricio Zapata-Rivera

**Affiliations:** 1 Escuela de Salud Pública, Universidad del Valle, Cali, Colombia Universidad del Valle Universidad del Valle Cali Colombia; 2 Departamento de Energía, Universidad de la Costa, Barranquilla, Colombia Corporación Universitaria de la Costa Universidad de la Costa Barranquilla Colombia

**Keywords:** conflictos armados, contaminación ambiental, salud ambiental, medición de riesgo, hidrocarburos policíclicos aromáticos, mercurio, minería, Armed conflicts, environmental pollution, environmental health, risk assessment, polycyclic aromatic hydrocarbons, mercury, mining

## Abstract

**Introducción.:**

Los conflictos armados afectan los territorios ricos en recursos y biodiversidad; el daño ambiental causado por las acciones violentas puede afectar la salud de las poblaciones.

**Objetivos.:**

Evaluar los riesgos para la salud humana debidos a la degradación ambiental asociada con tres acciones violentas en el marco del conflicto armado colombiano: la voladura de oleoductos, la minería informal con mercurio y la aspersión de cultivos ilícitos con glifosato.

**Materiales y métodos.:**

Se hizo una evaluación cuantitativa de los riesgos para la salud individual asociados con actividades del conflicto armado, usando metodologías que tienen en cuenta la ruta de dispersión de los contaminantes, su concentración en el ambiente, la exposición de los individuos y los riesgos de efectos cancerígenos y no cancerígenos.

**Resultados.:**

La evaluación de los riesgos asociados con las acciones en el marco del conflicto armado analizadas, evidenció un riesgo cancerígeno intolerable y uno no cancerígeno inaceptable debidos al consumo de agua y peces contaminados por hidrocarburos aromáticos policíclicos, mercurio y glifosato.

**Conclusiones.:**

El estudio reafirmó las conexiones inextricables que existen entre ambiente, sociedad y salud, y las implicaciones de la violencia ambiental para la salud pública de los grupos poblacionales vulnerables y, en general, para el bienestar de todos los seres vivos afectados por el conflicto armado.

## Abstract (en)

**Introduction:**

Armed conflicts affect territories rich in resources and biodiversity. As a result of the environmental damage caused by violent actions, the health of populations can be affected.

**Objectives:**

To assess the risks to human health due to environmental degradation associated with three violent actions in the context of the Colombian armed conflict: Pipeline bombing, informal mining with mercury, and spraying of illicit crops with glyphosate.

**Materials and methods:**

We conducted a quantitative evaluation of the risks to individual health associated with armed conflict activities using methodologies focused on the routes of pollutants dispersion, their concentrations in the environment, the exposure of the individuals, and the risks of carcinogenic and non-carcinogenic effects.

**Results:**

The risk assessment of the armed conflict-related actions under study evidenced intolerable carcinogenic risk and unacceptable non-carcinogenic risk due to the consumption of water and fish contaminated by polycyclic aromatic hydrocarbons (PAH), mercury, and glyphosate.

**Conclusions:**

The study reiterates the inextricable connections existing among the environment, society, and health, as well as the implications of environmental violence for the public health of vulnerable population groups and, in general, for the well-being of all living beings affected by the armed conflict.

Los conflictos armados ocurren con gran frecuencia en territorios ricos en recursos y biodiversidad. En los países del sur con economías extractivistas dependientes de la exportación de recursos primarios, la rica biodiversidad y la abundancia de recursos naturales a menudo resultan ser más una maldición que una bendición ^1^, pues las riquezas provenientes de la explotación de la naturaleza benefician solo a algunos, la competencia por los recursos genera conflictos socioambientales y, además, las ganancias obtenidas se utilizan para financiar la guerra, con un costo social y ecológico enorme para la mayoría de la población.

El daño ambiental causado por las acciones del conflicto armado puede afectar la salud de las poblaciones, pero los estudios sobre la degradación ambiental asociada con el conflicto y sus efectos en la salud humana son escasos, lo que limita la comprensión y la implementación de políticas y acciones durante los conflictos y después de ellos.

Según Zwijnenburg*, et al.,* las rutas directas de daño ambiental en un conflicto armado incluyen la contaminación por agentes químicos secundaria a los ataques contra sitios industriales, la contaminación por los residuos peligrosos de armas, y de los subproductos de las industrias extractivas usadas para financiar el conflicto, el daño a la infraestructura sanitaria, el desplazamiento de la población y la destrucción de los recursos naturales. Según estos autores, otros impulsores indirectos del deterioro ambiental son las debilidades de las economías en medio del conflicto y el colapso de la gobernanza ambiental de las autoridades estatales, el cual desemboca en el incumplimiento de las leyes y regulaciones ambientales ^2^.

A pesar de la firma del Acuerdo de Paz con la guerrilla de las FARC- EP en el 2016, después de más de cinco décadas de conflicto armado, Colombia es hoy uno de los países del mundo con mayor número de conflictos ambientales ^3^. Una de las acciones características del conflicto armado colombiano, llevada a cabo para afectar la industria extractiva y con un potencial efecto en la salud ambiental, ha sido la voladura de oleoductos, la cual produce incendios y contaminación por hidrocarburos. El petróleo es una mezcla compleja de compuestos hidrocarbonados que incluyen los hidrocarburos alifáticos (n-alcanos, isoalcanos y cicloalcanos), los hidrocarburos aromáticos policíclicos y sus derivados. El tipo de afectación sobre los organismos vivos depende de la ruta, la vía de exposición (ingestión, inhalación o dérmica) y del agente químico específico ^4^, pero siempre, las que primero reciben los efectos contaminantes en los ecosistemas son las especies de los niveles superiores de la red trófica.

Según el registro histórico del conflicto armado, en Colombia se han presentado eventos de voladura de oleoductos desde la década de 1960 y se estima que la infraestructura de transporte de hidrocarburos ha sido atacada 2.575 veces, y se han derramado cerca de 4,1 millones de barriles de petróleo ^5^^,^^6^, principalmente en los departamentos de Norte de Santander, Arauca, Putumayo y Nariño. Las poblaciones aledañas al estuario del río Mira, en el municipio de Tumaco, Nariño, se cuentan entre las más afectadas, como sucedió con el derrame de 410.000 galones de petróleo crudo causado por los atentados al oleoducto transandino en 2015 ^7^. Asimismo, a partir de la década de 1980, la zona de influencia del río Catatumbo se ha visto afectada por los derrames de petróleo a raíz de ataques contra el oleoducto Caño Limón-Coveñas. Desde entonces, más de 2,8 millones de barriles de petróleo han sido derramados en la zona, según el Ministerio de Ambiente y Desarrollo Sostenible ^8^.

Otra actividad adelantada en el marco del conflicto armado con efectos adversos en la salud ambiental, ha sido la minería informal de oro, en la cual se utiliza mercurio, y está en manos de agentes foráneos en territorios donde ancestralmente la practicaban comunidades campesinas como medio de subsistencia. La minería informal en Colombia alcanza entre el 50 y el 80 % de la actividad extractiva, se extiende en 65 % del territorio nacional y genera graves efectos ambientales por el deterioro de los ecosistemas, el agotamiento de la oferta ambiental y, en general, por la destrucción del entorno ^9^. Se ha informado, asimismo, que en estas zonas actúan intermediarios (con frecuencia grupos al margen de la ley) que financian la producción con antelación, lo que condiciona a los mineros a vender el oro a precios inferiores ^10^.

Las técnicas de extracción utilizadas en la minería informal con mercurio generan emisiones de material particulado, con consecuencias ocupacionales y ambientales; especialmente, la emisión de vapores de mercurio se produce al destilar la amalgama que el metal forma con el oro. El mercurio utilizado en las minas puede llegar, además, hasta los ecosistemas terrestres y acuáticos gracias a la escorrentía generada por el rebosamiento de las pocetas de cianuro o las malas prácticas en el manejo de este metal pesado. En los ecosistemas acuáticos, el mercurio se acumula y se magnifica en los peces que posteriormente son consumidos por el hombre y otras especies. Una vez que el mercurio ingresa al ambiente, forma metilmercurio, que es mucho más tóxico que el mercurio en su forma elemental ^11^.

Se estima que la cuenca alta del río Cauca, al suroccidente del país, se ve afectada por la actividad minera informal que se lleva a cabo en Suárez y otros municipios de los departamentos de Cauca y Valle del Cauca. Suárez es un municipio primordialmente minero, escenario de conflictos sociales debidos a los proyectos extractivos de empresas, inversionistas y grupos al margen de la ley ^12^. Los hechos violentos en este territorio han estado ligados a problemáticas de inequidad y exclusión potenciadas por un modelo de desarrollo económico que reproduce las brechas de desigualdad en el sector rural.

Otro sitio de minería informal en Colombia es la ciénaga de Ayapel, un complejo cenagoso de gran importancia ecológica, especialmente por la protección de aves acuáticas y migratorias, localizada en el departamento de Córdoba. Cerca de 650 hectáreas de esta ciénaga han sido arrasadas por la actividad minera y, según un informe de la Corporación Autónoma Regional de los Valles del Sinú y San Jorge ^13^ y otros estudios realizados en la zona ^14^, la concentración de mercurio en el agua de la ciénaga, consumida por los 50.000 habitantes del municipio de Ayapel, sobrepasa diez veces el contenido permitido por las organizaciones ambientales internacionales ^15^.

Por otra parte, en la lucha por los recursos que financian la guerra, la erradicación forzada de los cultivos de coca ha convertido la aspersión con glifosato en foco de controversia. Este es el herbicida de mayor uso a nivel mundial y una de las herramientas del actual modelo industrial de producción agrícola orientado a un incremento constante de la producción de alimentos ^16^. Entre 1974 y 2014, se aplicaron 9 billones de kilogramos de glifosato a nivel mundial, de los cuales dos terceras partes se utilizaron entre el 2004 y el 2014 ^17^. Este uso extensivo y creciente del glifosato ha generado un fuerte debate sobre sus efectos en el ambiente y la salud de las personas. Sin embargo, un análisis de la producción científica vinculada al glifosato entre 1974 y 2016 ^17^, demostró que durante los primeros treinta años los estudios se concentraron en el campo de las ciencias agrícolas y fueron financiados por las empresas productoras del herbicida como estrategia de posicionamiento comercial, lo que implica un conflicto de intereses. No obstante, en la última década se ha incrementado la producción científica independiente, y ha aumentado el interés por los estudios toxicológicos y los efectos ambientales. En 2015, el Centro Internacional de Investigaciones sobre Cáncer (*International Agency for Research on Cancer*, IARC) dio a conocer un informe en el que incluyó al glifosato en el grupo de las sustancias probablemente cancerígenas en humanos ^18^.

En Colombia, la aspersión aérea de marihuana con glifosato se inició en 1985 en la costa Atlántica, y se extendió a los cultivos de amapola y coca en 1992. La mezcla química usada para la aspersión tiene el nombre comercial de Roundup^®^ Ultra plus y está compuesta por glifosato, Cosmo-Flux^®^ 411F (aceite mineral y surfactantes no ionizados con agentes de acoplamiento) y el surfactante POEA (alquil-amina-polietoxilada), cuya acción determina un aumento de la toxicidad de este compuesto ^19^.

Según la Dirección Antinarcóticos de la Policía Nacional de Colombia (DIRAN), el uso del Roundup^®^ en la erradicación de cultivos ilícitos se ha extendido principalmente a cuatro regiones o núcleos en el país ^20^: Putumayo-Caquetá, Cauca-Nariño, Guaviare-Meta y Norte de Santander. Según la Oficina de las Naciones Unidas para las Drogas y el Crimen, las regiones de Putumayo-Caquetá (con 29.484 ha asperjadas) y Cauca-Nariño (con 57.897 ha) registran el mayor número y frecuencia de aspersiones con glifosato desde el 2001 ^21^. Esto coincide con el hecho de que, en el 2019, en estos departamentos, se localizaron seis de los diez municipios con mayor índice de amenaza para la diversidad cultural y biológica de Colombia, debido a las condiciones históricas asociadas con los cultivos de coca en cuanto al grado de afectación y la permanencia en el territorio: Tumaco y Barbacoas en Nariño, El Tambo en el Cauca, y Puerto Asís, Puerto Guzmán y Orito en Putumayo ^21^.

Según la Resolución 099 del 31 de julio de 2003 del Ministerio del Medio Ambiente, la dosis de glifosato para aspersión aérea de cultivos ilícitos en Colombia es de 10,4 L/hade la formulación comercial del herbicida glifosato conocida como Roundup^®^ 480 SL, más 0,25 L de Cosmo-Flux^®^ 411F y 13 litros de agua; esto representa una descarga de 23,65 litros de la mezcla por hectárea. En contraste, según la Resolución 1121 de 14 de septiembre de 2017 expedida por la Autoridad Nacional de Licencias Ambientales, la dosis de Roundup^®^ 480 SL para cultivos lícitos es de 2,0 L/ha, es decir que en la aspersión aérea de cultivos ilícitos se usa 5,2 veces la cantidad recomendada para otros cultivos, según los cálculos de Maldonado, *et al.*^22^. Esta dosificación se traduce en un incremento de la concentración de glifosato equivalente a 26 % comparada con el 1 % recomendado en Estados Unidos para aplicaciones terrestres, con equipos de protección y dirigido a malezas agrícolas.

Según los modelos tradicionales de análisis, para evaluar el riesgo para la salud debido a la exposición ambiental a estos contaminantes, se requiere caracterizar la fuente del contaminante, su dispersión, la exposición de la población y la dosis que ingresa al organismo. En estas evaluaciones, el riesgo es una función de la toxicidad de la sustancia peligrosa evaluada, y de la magnitud y el tiempo de exposición a ella ^23^.

En este contexto, se hizo una evaluación de riesgos para cuantificar los efectos en la salud de las poblaciones debidos a la degradación ambiental de los territorios asociada con el conflicto armado colombiano, en el marco de tres actividades estrechamente relacionadas con él: la voladura de oleoductos, la minería informal con mercurio y la aspersión de cultivos ilícitos con glifosato.

## Materiales y métodos

Se hizo una evaluación probabilística de los riesgos para la salud humana asociados con actividades del conflicto armado, usando la metodología estandarizada de la *U.S. Environmental Protection Agency* - USEPA (Agencia de Protección Ambiental de los Estados Unidos). Para la estimación cuantitativa de los riesgos de efectos cancerígenos y no cancerígenos, esta metodología tiene en cuenta la ruta de dispersión de los contaminantes, su concentración en el ambiente y la exposición de los individuos ^24^.

### Contaminantes evaluados, población y área de estudio

Se evaluaron los contaminantes ambientales representativos de cada una de las actividades analizadas del conflicto, una ruta y una vía de exposición de acuerdo con estudios recientes reportados en la literatura (cuadro 1). La población objetivo del estudio para todas las actividades evaluadas la conformaron 245 mujeres en edad fértil entre los 15 y los 40 años de edad, con condiciones sociales y económicas similares a las de las áreas de estudio. Este grupo se seleccionó por ser especialmente vulnerable frente a los eventos de contaminación ambiental y sus datos provenían de estudios similares desarrollados por Echeverry, *et al*. ^25^, y Zapata, *et al.*^26^. Las variables que se tuvieron en cuenta para las estimaciones de riesgo fueron: edad, tiempo de exposición, duración de la exposición, peso corporal, cantidad de pescado y agua consumidos por ración, frecuencia de consumo y tiempo promedio, este último calculado según las recomendaciones de la USEPA ^24^.

**Cuadro 1:** Contaminantes, ruta, vía de exposición y región de las actividades evaluadas del conflicto armado colombiano

Actividad	Contaminantes	Ruta de exposición	Vía de exposición	Región
Voladura de oleoductos	HAP: naftaleno, pireno y criseno	Consumo de pescado Consumo de agua	Ingestión	Estuario del río Mira, costa sur del municipio de Tumaco, Nariño (6) Río Catatumbo, Norte de Santander ^25^
Contaminación por uso de mercurio en la minería informal	Mercurio	Consumo de pescado	Ingestión	Río Cauca entre el corregimiento de El Hormiguero en Cali y la vereda Paso de la Torre en Yumbo, Valle del Cauca ^23^
Erradicación forzada de cultivos ilícitos con fumigaciones aéreas de glifosato	Glifosato y AMPA	Consumo de agua	Ingestión	Ciénaga de Ayapel, Córdoba ^26^ Departamentos de Putumayo, Caquetá, Cauca y Nariño ^28^

HAP: hidrocarburos aromáticos policíclicos

Las áreas de estudio corresponden a zonas del territorio colombiano donde suelen ocurrir los tres tipos de acciones características del conflicto armado: la voladura de oleoductos, la minería informal con mercurio y la aspersión con glifosato para la erradicación forzada de cultivos ilícitos. Su selección se justifica, además, por la disponibilidad de datos de las variables necesarias para hacer el cálculo de los riesgos. En la evaluación de los efectos de la voladura de oleoductos, se seleccionó la región del río Mira, y se incluyeron los hidrocarburos aromáticos policíclicos naftaleno, pireno y criseno, por ser tres de los compuestos más nocivos para la salud ^27^. Sobre estos últimos se dispone de los datos de la cuantificación hecha por Garcés, *et al.,* dos años después de los atentados al oleoducto transandino de junio del 2015 ^7^; en peces, las concentraciones de naftaleno, pireno y criseno fueron de 0,1095, 5,6264 y 18,0088 mg/kg, respectivamente. Los valores utilizados para la frecuencia de exposición al consumo de pescado estuvieron entre 40,5 y 162,0 días/año, con un promedio de 94,6 (desviación estándar, DE=4,32x10^1^ días/año). Además, se incluyó la región del Catatumbo, utilizando la suma total de hidrocarburos calculada por la Corporación Autónoma de Norte de Santander entre el 2017 y el 2018 ^28^. La concentración promedio de hidrocarburos aromáticos policíclicos en aguas del río correspondió a 2,8 mg/L y el tiempo promedio de exposición fue de 34 años.

En cuanto a la minería informal con mercurio, la ruta de exposición seleccionada fue la ingestión de peces provenientes del río Cauca a su paso por el Valle del Cauca, así como de la ciénaga de Ayapel, en tanto que la especie química evaluada del mercurio fue el metilmercurio, pues más del 90 % de este metal se presenta en esta forma en la biota acuática ^29^. Las cuantificaciones se tomaron de los estudios de Zapata*, et al.*^26^^,^^30^ mercurio (Hg, en el río Cauca entre 2014 y 2015, y el de Marrugo, *et al.*^29^, en la ciénaga de Ayapel entre 2004 y 2005. Las concentraciones utilizadas estuvieron entre 0,79 x 10^-1^ y 4,90 x 10^-1^ mg/kg (promedio: 2,39 x 10^-1^) en peces del río Cauca y entre 0,35 x 10^-1^ y 6,5 x 10^-1^ mg/kg (promedio: 3,54 x 10^-1^) en la ciénaga de Ayapel, la exposición se produjo durante toda la vida de las mujeres, es decir, su duración promedio equivalía a la edad promedio de ellas.

Asimismo, para las concentraciones de glifosato y ácido aminometilfosfónico (AMPA) en agua, se tomaron como referencia los datos obtenidos de los estudios de Silva, *et al.,* quienes, en un ecosistema similar al colombiano y con un número adecuado de muestras, determinaron la contaminación de aguas por uso de glifosato mediante cromatografía líquida de alta resolución, 30 a 60 días después de la aplicación del herbicida en cultivos lícitos entre el 2001 y el 2002 ^31^. Como en estas mediciones no se tuvo en cuenta la mezcla química usada para la aspersión de cultivos ilícitos en Colombia, compuesta por glifosato, Cosmo-Flux^®^ 411F y el surfactante POEA, y tampoco, que en esta se utilizan concentraciones cinco veces más altas que las de uso agrícola convencional, se utilizó este valor como un factor multiplicador común en cada estimación por departamento. Así, utilizando concentraciones de base por departamento a partir de la información de la cantidad de aspersiones en cada uno de ellos (desde 0,03 mg/kg en el Cauca hasta 0,10 mg/kg en Nariño), la concentración final utilizada fue cinco veces ese valor inicial, entre 0,15 mg/kg en el Cauca y 0,50 mg/kg en Nariño, valores que se consideraron basales (“escenario de base”). Para estimar la duración de la exposición, se tuvieron en cuenta los registros históricos de fumigación de cultivos ilícitos, según los cuales dicha duración sería de 15 años en todos los departamentos de la zona evaluada (entre el 2001 y el 2015), excepto en Cauca, donde sería un año menos, pues no se reportaron fumigaciones en el 2002 ^32^.

Además de este llamado “escenario de base”, se simuló una segunda situación que diera cuenta de los fenómenos de bioacumulación y biomagnificación del glifosato en la red trófica. En diversos estudios recientes, se ha comprobado que el glifosato se acumula y magnifica en seres vivos como los peces y las plantas, y se distribuye ampliamente en distintos compartimentos ambientales en forma de material particulado suspendido en los cuerpos de agua, los sedimentos, el suelo y la cobertura vegetal ^33^^-^^36^. Se ha demostrado que el factor de bioacumulación (*bioaccumulation factor*, BAF) del glifosato en estas matrices ambientales puede alcanzar un rango entre 0,5 y 5 ^34^^,^^37^. En consecuencia, para dar cuenta de estos fenómenos, se multiplicó por tres el valor de base (primer escenario) como referente del valor promedio de los BAF reportados. En Colombia, no se cuenta con estudios rigurosos que permitan conocer experimentalmente los fenómenos derivados de la aspersión de cultivos ilícitos, razón por la cual se estimó conveniente hacerlo de esta forma.

### Dosis y estimaciones de riesgo

En el cálculo de la dosis interna de los diferentes contaminantes después de la exposición a la contaminación, se utilizó la siguiente ecuación recomendada por la USEPA ^24^:




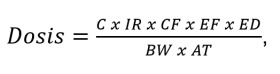




donde C corresponde a la concentración en la muestra (mg.kg^-1^), IR a la tasa de ingestión de la muestra (mg.día^-1^), CF al factor de conversión (1 x 10^-6^ kg.mg^-1^), EF a la frecuencia de exposición (días.año^-1^), ED a la duración de la exposición (años), BW al peso corporal (kg), y AT al tiempo promedio (días)

Con base en esta dosis, se evaluaron los riesgos con respecto a un marcador de toxicidad característico de cada contaminante. Para los riesgos de efectos no cancerígenos, se usó la dosis de referencia (*reference dose*, RfD), o máxima dosis oral aceptable de una sustancia tóxica, de los contaminantes evaluados en el cuadro 2. El cálculo de riesgos no cancerígenos se hizo utilizando el cociente entre la dosis y la Rfd, o cociente de riesgo (*hazard quotient*, HQ). Si el valor estimado del HQ es mayor de 1, se considera que el riesgo para la salud es inaceptable ^38^.

**Cuadro 2:** Valores de dosis de referencia y factor de pendiente del cáncer de los contaminantes incluidos en la evaluación de riesgos en salud

Contaminante	RfD mg/kg-día	CSF mg/kg-día	Referencia
Naftaleno	2,00 x 10-^2^	1,20 x 10-^1^	USEPA, 1998
Pireno	3,00 x 10-^4^	1,00 x 10-^1^	USEPA, 2017
Criseno	ND	1,00 x 10-^3^	Michigan State, 2015
HAP Totales	3,00 x 10-^4^	1,00 x 10-^1^	Bulder, et al., 2006
Metilmercurio	1,00 x 10-^4^	3,50 x 10-^2^	USEPA, 1987
Glifosato	1,00 x 10-^1^	ND	USEPA, 1987
AMPA	3,00 x 10-^1^	ND	Minnesota Dep. of Health, 2017

RfD: dosis de referencia; CSF: factor de pendiente del cáncer; ND: no disponible; HAP: hidrocarburos aromáticos policíclicos

Según la literatura científica, la exposición a dos o más contaminantes puede causar efectos aditivos o interactivos debido a la sinergia entre estos. En estos casos, se recomienda estimar el cociente de riesgo combinado (*combined* HQ, CHQ), el cual se calculó para la exposición a glifosato más AMPA, siguiendo las recomendaciones de Miri, *et al*. ^39^.

Complementariamente, el riesgo cancerígeno se estimó a partir del producto entre la dosis calculada y el factor de pendiente del cáncer (*Cancer Slope Factor*, CSF), que corresponde al límite superior del intervalo de confianza del 95 % del incremento del riesgo de cáncer asociado con la exposición durante la vida por ingestión o inhalación de un agente; los valores de los CSF se muestran en el cuadro 2. Un nivel de riesgo cancerígeno estimado de 1 x 10^-N^ indica que hasta un caso de cáncer por cada 10^N^ habitantes puede ser producto de causas ajenas al factor evaluado (por ejemplo, cigarrillo, radiación, etc.); los casos por encima de dicho umbral se atribuyen a la variable bajo estudio. En general, se acepta que valores de riesgo cancerígeno inferiores a 1 por cada 100.000 habitantes (1 x 10^-5^) se consideran tolerables, en tanto que los riesgos mayores de 1 por 10.000 habitantes (1 x 10^-4^) son intolerables. Las estimaciones entre estos dos valores (1 x 10^-5^ y 1 x 10^-4^) se consideran en el rango que amerita medidas de prevención ^40^^-^^42^. Es de resaltar que no se dispone de valores del CSF para el glifosato y su metabolito, el AMPA, lo que imposibilita en este caso la estimación de ese riesgo potencial.

El cálculo probabilístico del riesgo implica un muestreo aleatorio de cada una de las variables involucradas en la exposición para la estimación de la dosis según la ecuación anterior ^30^. Con el objetivo de construir las distribuciones probabilísticas de cada una, se utilizó el análisis de Monte Carlo, uno de los métodos probabilísticos más utilizados con este fin ^23^. En consecuencia, para el cálculo del riesgo, se siguieron las directrices del programa computacional Crystal Ball 11.0® (Decisioneering Inc., Denver, Colorado, EUA) con simulaciones de tipo Monte Carlo para 100.000 iteraciones basadas en los tipos de distribución de probabilidad de cada variable. Para ello, se utilizó la prueba de normalidad de Shapiro Wilks, empleando el programa computacional Statistica, v. 8.0 (StatSoft, Poland). Posteriormente, se determinó el ajuste de los datos a la distribución mediante la prueba de ji al cuadrado.

## Resultados

En el cuadro 3, se presentan los valores del riesgo cancerígeno y no cancerígeno en mujeres en edad fértil por consumo de agua en el río Catatumbo y pescado en el río Mira. Los resultados demuestran que el contenido de hidrocarburos aromáticos policíclicos totales y de algunos de sus compuestos específicos, especialmente el pireno, representan riesgos cancerígenos intolerables y riesgos no cancerígenos inaceptables para la población. Se estarían presentando entre dos y ocho casos de cáncer por cada 1.000 personas expuestas a hidrocarburos aromáticos policíclicos totales en la zona de influencia del río Catatumbo o, lo que es lo mismo, entre 225 y 846 casos por cada 100.000 expuestos. En cuanto al consumo de peces con pireno, el riesgo cancerígeno podría afectar hasta a 111 personas por cada 100.000 expuestos en la zona de influencia del río Mira. Todos estos valores de riesgo son intolerables, pues son mucho mayores que el valor máximo de un caso por 100.000 personas.

**Cuadro 3:** Valores del riesgo cancerígeno y no cancerígeno asociado con la exposición a hidrocarburos en mujeres en edad fértil por consumo de agua del río Catatumbo y pescado del río Mira

Estadísticas	Río Catatumbo Consumo de agua		Río Mira Consumo de pescado				
	Riesgo cancerígeno	Riesgo no cancerígeno	Riesgo cancerígeno			Riesgo no cancerígeno	
	HAP totales		Criseno	Naftaleno	Pireno	Naftaleno	Pireno
Mínimo	2,25 x 10^-3^	7,50	3,64 x 10^-7^	7,03 x 10^-7^	1,13 x 10^-5^	1,11 x 10^-4^	0,38
Máximo	8,46 x 10^-3^	28,1	3,55 x 10^-5^	6,28 x 10^-6^	1,11 x 10^-3^	1,08 x 10^-2^	37,0
Coefciente de variación	0,3120				0,6430		
Error estándar medio	4,18 x 10^-^6	1,39 x 10^-2^	2,16 x 10^-8^	1,16 x 10^-8^	6,76 x 10^-7^	6,57 x 10^-6^	2,25 x 10^-2^

HAP: hidrocarburos aromáticos policíclicos

Riesgo cancerígeno: Tolerable: p<1x10-^5^

Rango de medidas de prevención: p1x10-^5^ <p<1x10-^4^ (negrilla)

Intolerable: p>1x10-^4^ (en rojo)

Riesgo no cancerígeno (HQ): >1 inaceptable (en rojo)

Por otra parte, el riesgo no cancerígeno estimado en el agua del Catatumbo sugiere que la dosis alcanzada puede ser hasta 28 veces superior a la máxima aceptada para los hidrocarburos aromáticos policíclicos totales y, en peces del río Mira, hasta 37 veces por encima de la máxima aceptada de pireno. Es importante tener en cuenta, además, que en esta evaluación de riesgo solo se incluyeron tres hidrocarburos aromáticos policíclicos de los cien tipos distintos identificados, por lo que la exposición y sus efectos en la salud pueden ser aún mayores.

En cuanto a la exposición a mercurio por el consumo de pescado contaminado a causa de las actividades de minería informal, las estimaciones de los riesgos cancerígenos máximos sobrepasaron los valores tolerables tanto en el río Cauca como en la ciénaga de Ayapel y estuvieron en el rango de los valores que requieren prevención: esto es, aproximadamente entre 3 y 5 casos por cada 100.000 expuestos (cuadro 4). Se estimó, asimismo, que en el río Cauca la dosis de mercurio ingerida por los peces podría llegar a ser hasta 9,3 veces la máxima aceptable para ser no cancerígena. En otras palabras, entre los valores de riesgo probables, la estimación incluyó aquellos por encima de lo tolerable que ameritan intervenciones de remediación. En Ayapel, los valores estimados estuvieron por debajo del máximo aceptable (menores de 1).

**Cuadro 4:** Valores del riesgo cancerígeno y no cancerígeno asociado con la exposición a mercurio en mujeres en edad fértil por consumo de pescado del río Cauca y la ciénaga de Ayapel

Estadísticas	Río Catatumbo Consumo de gua		Río Mira Consumo de pescado	
	Riesgo cancerígeno	Riesgo no cancerígeno	Riesgo cancerígeno	Riesgo no cancerígeno
	Mercurio		Mercurio	
Mínimo	8,03 x 10^-9^	2,29 x 10-^3^	4,01 x 10-^9^	1,14 x 10-^3^
Máximo	3,26 x 10^-5^	9,32	4,55 x 10-^5^	0,130
Coeficiente de variación	1,07		1,14	
Error estándar medio	7,74 x 10-^9^	2,21 x 10-^3^	9,85 x 10-^9^	2,82 x 10-^3^

Riesgo cancerígeno: Tolerable: p<1 x 10-^5^

Rango de medidas de prevención: p1x10-^5^ <p<1x10-^4^ (negrilla)

Intolerable: p>1x10-^4^ (en rojo)

Riesgo no cancerígeno (HQ): >1 inaceptable (en rojo)

Por último, en condiciones con valores basales, ninguna de las evaluaciones de riesgo no cancerígeno del glifosato y el AMPA mostró valores por encima de los máximos aceptables (cuadro 5). En cambio, en la simulación de bioacumulación y biomagnificación del agente en la red trófica, se identificaron valores por encima del máximo aceptable para consumo de agua contaminada con glifosato en el departamento de Nariño. Cuando se estimaron los cocientes de riesgo combinados para glifosato y AMPA, los valores de riesgo estimados excedieron el máximo aceptable en Nariño y Putumayo (cuadro 6).

**Cuadro 5:** Valores del riesgo no cancerígeno asociado con la exposición a glifosato y AMPA en mujeres en edad fértil por consumo de agua en áreas asperjadas de Colombia

Estadísticas	Nariño	Putumayo	Caquetá	Cauca	Nariño	Putumayo	Caquetá	Cauca
	Riesgo no cancerígeno por consumo de agua							
	Glifosato				AMPA			
Mínimo	0,145	0,101	0,129	0,044	0,048	0,034	0,0430	0,014
Máximo	0,460	0,321	0,149	0,138	0,153	0,107	0,0497	0,046
Coeficiente de variación	0,3004	0,3005	0,0418	0,3000	0,3002	0,3004	0,0419	0,2996
Error estándar medio	2,35 x 10-4	1,65 x 10-4	1,84 x 10-5	7,06 x 10-5	7,86 x 10-5	5,51 x 10-5	6,14 x 10-6	2,35 x 10-5

Los cocientes de riesgo combinado para glifosato más AMPA por departamento, fueron: Nariño, 0,613; Putumayo, 0,428; Caquetá, 0,199; Cauca, 0,184

**Cuadro 6:** Valores del riesgo no cancerígeno en mujeres en edad fértil asociado con la exposición a glifosato y AMPA por consumo de agua en áreas asperjadas de Colombia. Caso 2: bioacumulación y biomagnificación del glifosato en la red trófica

Estadísticas	Nariño	Putumayo	Caquetá	Cauca	Nariño	Putumayo	Caquetá	Cauca
	Riesgo no cancerígeno por consumo de agua							
	Glisofato				AMPA			
Mínimo	0,435	0,304	0,387	0,130	0,146	0,101	0,129	0,044
Máximo	1,370	0,964	0,448	0,413	0,150	0,106	0,149	0,138
Coeficiente de variación	0,2997	0,3002	0,0418	0,2999	0,8950	0,9010	0,1260	0,8991
Error estándar medio	7,06 x 10-4	4,94 x 10-4	5,52 x 10-5	2,12 x 10-4	2,36 x 10-4	1,65 x 10-4	1,84 x 10-5	7,05 x 10-5

Los cocientes de riesgo combinado para glifosato más AMPA por departamento, fueron: Nariño, 1,52; Putumayo, 1,07; Caquetá 0,60; Cauca 0,55

Riesgo no cancerígeno (HQ): >1 inaceptable (en rojo)

## Discusión

Esta evaluación de los riesgos de tres tipos de acciones relacionadas con el conflicto armado en Colombia, evidenció riesgo cancerígeno intolerable y riesgo no cancerígeno inaceptable, por consumo de agua y peces contaminados por hidrocarburos aromáticos policíclicos, mercurio y glifosato. Los hallazgos concuerdan con otras evaluaciones de riesgo de estos mismos contaminantes, pero en situaciones asociadas con actividades humanas no enmarcadas en el conflicto armado. En el presente estudio, las acciones de violencia ambiental relacionadas con el conflicto corresponden a actividades productivas en las que diversos actores armados han sido los presuntos responsables: las guerrillas, en el caso de la voladura de oleoductos; principalmente los paramilitares, en el caso de la minería de oro informal, y el Estado, en el caso de la aspersión con glifosato para la erradicación forzada de cultivos ilícitos.

En el caso de la voladura de oleoductos, los modelos evidenciaron un intolerable riesgo cancerígeno debido al consumo de agua contaminada por hidrocarburos aromáticos policíclicos en el río Catatumbo y al consumo de pescado del río Mira contaminado con pireno, uno de los compuestos más nocivos sobre los que se dispone de información. En estos dos casos, el riesgo no cancerígeno también resultó inaceptable al superar entre siete y más de 30 veces la dosis máxima aceptable. Una evaluación de riesgos más exhaustiva podría incluir la cuantificación de otros hidrocarburos aromáticos policíclicos, y otras rutas y vías de exposición (por ejemplo, ingestión, absorción dérmica e inhalación) que aumentarían aún más la probabilidad de efectos nocivos para la salud ^23^.

Los resultados demostraron categóricamente un aumento en el riesgo de cáncer y otras afectaciones de la salud de estas poblaciones. Según la IARC, la exposición a hidrocarburos aromáticos policíclicos en la dieta se ha asociado específicamente con un aumento del riesgo de adenoma colorrectal y cáncer pancreático en humanos, en tanto que otras rutas de exposición, por ejemplo la inhalación o el contacto dérmico, así como los estudios en modelos animales, han demostrado el aumento del riesgo de cáncer de pulmón y de otros órganos ^43^.

Por otra parte, la *Agency for Toxic Substances and Disease Registry* (ATSDR) incluye diversos problemas de salud reproductiva, defectos de nacimiento y bajo peso al nacer, además de efectos nocivos en la piel y en los fluidos corporales, y disminución en la habilidad para combatir infecciones, después de exposiciones de corta o larga duración a hidrocarburos aromáticos policíclicos con posibles efectos no cancerígenos para la población ^44^.

A la luz de los resultados de la presente evaluación probabilística de riesgos en salud por exposición a hidrocarburos aromáticos policíclicos, llaman la atención los análisis realizados dentro del área de influencia de estas exposiciones en Tumaco, que mostraron una gran prevalencia de leucemia, de tumores benignos o de evolución incierta, de tumores malignos de estómago, de tumores malignos de tráquea o bronquios y de otros, asociados con un exceso de años de vida potencialmente perdidos por muerte prematura, especialmente en mujeres ^45^, lo cual podría potenciarse en el contexto de estas regiones por el difícil acceso a un diagnóstico oportuno y a tratamientos especializados.

Por otra parte, se estimó un aumento en el riesgo no cancerígeno por consumo de pescado del río Cauca contaminado con mercurio debido a las actividades de minería informal, con coeficientes de riesgo que podrían alcanzar hasta más de nueve veces el valor máximo tolerable. Asimismo, tanto en el río Cauca como en la ciénaga de Ayapel, los riesgos cancerígenos estimados por exposición al mercurio están en el rango de los valores que ameritan medidas de prevención, pues son superiores a un caso por 100.000 expuestos. El mercurio es un neurotóxico muy conocido que puede afectar el desarrollo fetal y durante la infancia, así como causar la aparición o exacerbación de déficits neurológicos en adultos mayores previamente expuestos ^46^^-^^48^. En cuanto a los efectos cancerígenos del mercurio, se ha descrito la asociación con adenomas renales, adenocarcinomas y otros tipos de carcinomas y, en general, su capacidad de producir daño cromosómico y nuclear ^49^.

En este sentido, en estudios de campo en el municipio de Ayapel (Córdoba), se evidenciaron concentraciones de mercurio total en cabello (valores promedio estimados: 2,18 ± 1,77 μg/g) superiores a las permitidas internacionalmente por la USEPA (1 μg/g) en población mayor de 14 años ^14^. Asimismo, síntomas como cefalea, irritabilidad, falta de concentración, insomnio, alteración de la presión arterial, sabor metálico, úlceras bucales, parálisis facial, náuseas y hormigueo de las manos, se han asociado con los niveles de mercurio cuantificados, que se deben, presumiblemente, al elevado consumo de pescado de la ciénaga de Ayapel contaminado con mercurio ^14^. Por otra parte, en la zona de influencia del río Cauca al oriente de la ciudad de Cali, incluida en este análisis de evaluación de riesgos, los estudios de campo han demostrado una mayor prevalencia de malformaciones congénitas en neonatos, por ejemplo, la sirenomelia, con una frecuencia de tres casos por 1.000 nacimientos, cuando lo esperado para estas malformaciones es de uno por 100.000 nacimientos ^50^^,^^51^.

Por último, en condiciones basales la evaluación de riesgo no cancerígeno de la aspersión con glifosato no registró ningún valor por encima del máximo tolerable para la exposición al glifosato o a su metabolito AMPA, aisladamente o en combinación (todos los cocientes de riesgo combinados fueron menores de uno). No obstante, al evaluar la bioacumulación y la biomagnificación de estos agentes en la red trófica, se obtuvieron valores de riesgo por encima de los máximos permitidos para la exposición al glifosato, solo o en combinación con AMPA, en los departamentos de Nariño y Putumayo. En este sentido, los efectos del glifosato, especialmente en la salud reproductiva (fertilidad), el desarrollo fetal o el desarrollo posnatal por exposición *in útero*, se han documentado bastante en estudios de laboratorio y en modelos animales ^52^^-^^55^, aunque en los estudios epidemiológicos en humanos persiste el debate sobre sus efectos ^56^^-^^58^.

A pesar de su reclasificación como probablemente cancerígeno para los humanos (grupo 2) por parte de la IARC, también hay bastante controversia sobre los potenciales efectos cancerígenos del glifosato, por lo cual no existe un valor de CSF avalado internacionalmente. A pesar de ello, con base en el CSF reportado por el estado de California en Estados Unidos, CSF=0,00062 x 10^-1^ mg/kg/día ^59^, se estimaron los valores del riesgo cancerígeno por exposición al glifosato, los cuales fueron mayores de 1 x 10^-5^ en todos los departamentos (los cálculos no se muestran); las mayores estimaciones de riesgo (2,27 a 6,25 x 10^-5^) se obtuvieron en Nariño sin tener en cuenta el AMPA, lo que sugiere que es necesario tomar medidas de prevención.

El estudio presenta limitaciones por la falta de más y mejores datos sobre las exposiciones, de información proveniente de programas sistemáticos de muestreo para determinar las concentraciones y, en general, de un mayor y más riguroso seguimiento de las condiciones de las matrices ambientales que son fuente de exposición en estas poblaciones vulnerables. No obstante, los estudios utilizados como base para la evaluación del riesgo son confiables, y nos ayudan a comprender la magnitud del impacto del conflicto armado como causa de contaminación y riesgo ambiental.

La situación de riesgo descrita se agrava aún más cuando se considera el contexto de vulnerabilidad de estos territorios, sobre todo si se tiene en cuenta el deficiente acceso a agua potable en las zonas rurales más afectadas por el conflicto armado. La cobertura promedio de acueducto en zonas rurales del país en el 2017 era de 73,2 % y los índices de calidad del agua indicaban que el 58 % de la población rural recibía agua no apta para el consumo ^60^. Además, algunos de los territorios evaluados aquí tienen coberturas de prestación del servicio de acueducto incluso más bajas, como en el caso de Tumaco, donde solo el 51 % de los habitantes tienen cobertura, o en la zona del Catatumbo donde, según la Defensoría del Pueblo, solo el 27 % de la población tiene acceso a agua potable ^8^. Sumado a esto, la concentración de la pobreza en las regiones afectadas por el conflicto, por ejemplo en el Catatumbo, se refleja en que más del 53 % de la población allí se encuentra bajo la línea de pobreza ^8^, lo que seguramente favorece sinergias negativas en salud dado el déficit nutricional de las poblaciones expuestas.

Asimismo, en algunas de estas comunidades, específicamente la exposición por consumo de pescado se ve favorecida porque este alimento hace parte de los hábitos nutricionales para su sustento, de su cultura y de sus modos de vida. Por esto, no se debe adoptar intervenciones enfocadas en restringir el consumo de pescado, sino en la mitigación o biorremediación de la concentración de los contaminantes en el ambiente, en el fortalecimiento de la seguridad y la soberanía alimentarias y, en general, en el mejoramiento de las condiciones de vida de la población expuesta. Además, es necesario implementar acciones de monitoreo de la contaminación en estos y otros sitios con problemas similares por las acciones de violencia ambiental asociadas con el conflicto armado.

En conclusión, este estudio reafirma las conexiones inextricables que existen entre ambiente, sociedad y salud, y la urgencia de consolidar las acciones en pro de una paz duradera y sostenible, dadas las implicaciones que ello tiene para la salud pública de estos grupos poblacionales vulnerables y el bienestar de todos los seres vivos afectados por el conflicto armado.
